# Behavioural responses of females of two anopheline mosquito species to human-occupied, insecticide-treated and untreated bed nets

**DOI:** 10.1186/1475-2875-13-294

**Published:** 2014-07-30

**Authors:** James F Sutcliffe, Shaoman Yin

**Affiliations:** 1 Department of Biology, Trent University, Peterborough, Ontario K9J 7B8, Canada; 2 Entomology Branch, US Centers for Disease Control and Prevention, Atlanta, Georgia 30329, USA; 3 Department of Biostatistics and Bioinformatics, Emory University, Atlanta, Georgia 30322, USA

**Keywords:** *Anopheles*, Host-seeking, Bed nets, Durability, ITNs, LLINs

## Abstract

**Background:**

Insecticide-treated bed nets (ITNs), used extensively to reduce human exposure to malaria, work through physical and chemical means to block or deter host-seeking mosquitoes. Despite the importance of ITNs, very little is known about how host-seeking mosquitoes behave around occupied bed nets. As a result, evidence-based evaluations of the effects of physical damage on bed net effectiveness are not possible and there is a dearth of knowledge on which to base ITN design.

**Methods:**

The dispersion of colony-raised female *Anopheles gambiae* and *Anopheles albimanus* was observed in 2-hr laboratory experiments in which up to 200 mosquitoes were released inside a mosquito-proof 3 m × 3 m tent housing a bed net arrayed with 18 30 cm × 30 cm sticky screen squares on the sides, ends and roof. Numbers of mosquitoes caught on the sticky squares were interpreted as the ‘mosquito pressure’ on that part of the net.

**Results:**

Presence of a human subject in the bed net significantly increased total mosquito pressure on the net for both species and significantly re-oriented *An. gambiae* to the roof of the net. *Anopheles albimanus* pressure was greatest on the bed net roof in both host-present and no-host conditions. The effects of different human subjects in the bed net, of different ambient conditions (dry, cool conditions *vs* warm, humid conditions) and of bed net treatment (deltamethrin-treated or no insecticide) on mosquito pressure patterns were tested for both species. Species-specific pressure patterns did not vary greatly as a result of any of these factors though some differences were noted that may be due the size of the different human subjects.

**Conclusions:**

As a result of the interaction between host-seeking responses and the convective plume from the net occupant, species-specific mosquito pressure patterns manifest more or less predictably on the bed net. This has implications for bed net design and suggests that current methods of assessing damaged bed nets, which do not take damage location into account, should be modified.

## Background

Insecticide-treated bed nets (ITNs), increasingly in the form of long-lasting, insecticide-treated bed nets (LLINs) in which insecticide is incorporated in the net material at the time of manufacture, have become a mainstay of international efforts to reduce the burden of malaria with hundreds of millions having been distributed by programmes such as The President’s Malaria Initiative, Rollback Malaria, Against Malaria Foundation, the Global Fund and many others. ITNs provide a dual line of defence against night-biting, potentially malaria-infected anopheline mosquitoes. The first line of defence is the physical barrier any bed net presents to mosquitoes. This protection is highly effective when the bed net is intact and properly used [1,2] but is quickly compromised when the untreated net develops rips and tears [3]. The second line of defence, a pyrethroid insecticide in the netting material, kills or knocks down susceptible mosquitoes generally after periods of contact of several minutes [4,5]. Ideally, this occurs before the mosquito enters the net and bites but it may also occur when the mosquito lands on the inside of the bed net after biting the occupant. In the former case, the possibility of malaria transmission to or from the occupant is completely negated. In the latter case, an infected mosquito may transmit malaria to the net occupant but it will not pass it any further and a mosquito that is infected by biting an infected person inside the ITN will not survive to reproduce or to transmit malaria. Insecticides in ITNs also exert various behavioural effects on mosquitoes. Depending on the insecticide and mosquito species, these effects may result in reduced house entry [6-8], reduced blood feeding success [6] and greater likelihood of house exit [6]. In addition, high rates of ITN use in some communities have been associated with a generalized reduction in malaria vector incidence for some distance beyond the communities themselves [9]. The mechanisms and implications of these effects are not fully understood. They may be positive if they reduce the number of mosquitoes entering bed nets that are otherwise vulnerable due to rips and tears [3,10]. Negative consequences have also been ascribed to some of these behavioural effects since they may prevent mosquitoes from landing on ITN material long enough to pick up a lethal dose of insecticide [11-13].

Given the large number of ITNs in use and the importance of this strategy to the overall goals of malaria reduction and control, effective ways of monitoring ITN condition over time are needed so that decisions that maximize desired public health outcomes in economically sustainable ways can be made about when to replace deteriorating cohorts of ITNs. Accordingly, much effort has been put into developing methods to monitor ITN integrity. These methods take the form of bioassays such as the cone test and tunnel tests [14] that determine insecticide lethality and knockdown capabilities, and chemical assays employing methods such as gas chromatography and HPLC to determine residual amounts of insecticide in aging bed nets [15]. Comparison of these results to known or determined critical levels of insecticide needed to achieve knock-down, excito-repellency, etc., of endemic populations of vector species can then be used to determine ITN insecticide efficacy.

Many programmes also collect information on ITN physical condition and deterioration over time. Some of these data, combined with data on insecticide content, have proven alarming since they suggest that both chemical and physical aspects of some LLINs are deteriorating faster than anticipated in some settings [16,17]. Unfortunately however, although a great deal of information about ITN physical condition has been collected, there is as yet no evidence-based way to assess how the specifics of hole size, location and shape affect bed net effectiveness, i.e., how they affect the probability of mosquito entry into the human-occupied bed net.

ITNs have been evaluated mainly in terms of human factors and factors humans can control such as net cost, shape and size, choice of insecticide, distribution logistics, durability, community acceptance, usage, attrition, etc. While these are important considerations, there is another less commonly adopted perspective that is also important – the mosquito’s. For the mosquito, the ITN is an obstacle like many others that its host-seeking ‘programme’ must respond to while it is attempting to get a blood meal. Mosquitoes, like other host-seeking Diptera, orient to their hosts from different distances using a wide range of host and environmental cues [18-20] which ultimately define species’ host preferences, preferred biting sites on the host, activity periods and so-on. Of particular relevance in the ITN context is how stimuli that mediate host seeking at close range affect different *Anopheles* species. For instance, DeJong and Knols [21] found that *Anopheles gambiae s.s*. and *Anopheles atroparvus*, respectively anthropophilic and generalist feeders, respond differently to CO_2_, moisture and heat by biting a human test subject on different parts of the body. *Anopheles atroparvus* oriented strongly to the CO_2_ and breath odours biting predominantly around the face and upper body while *An. gambiae* ignored breath and CO_2_, appearing instead to move down the body along convective currents to bite the lower extremities. Differential responses to close range cues were also shown by Dekker *et al.*[22] to be at play in determining preferred biting sites for each of *An. gambiae s.s*., *Anopheles arabiensis* and *Anopheles quadriannulatus*, all members of the same species complex. Given the great variety seen in responses to close range cues even in closely related mosquitoes and given that this variety must then interact with the human-occupied ITN, there is every reason to expect that patterns of mosquito behaviour around ITNs will also vary with mosquito species and other factors and that these behavioural patterns could have implications for ITN effectiveness under various circumstances of physical and insecticide deterioration. Despite this, but for the important recent exception of Lynd and McCall’s work on *An. gambiae*[23], there is little understanding of how any *Anopheles* species responds to the occupied bed net nor is anything known about how these responses interact with ITNs as they deteriorate both physically and chemically. To help to address this deficit, an examination of host-seeking mosquito orientation to, and dispersion around, human-occupied intact bed nets in a laboratory setting was undertaken. The aim of this study was to determine how mosquitoes distribute themselves around the intact human-occupied bed net since the resulting ‘mosquito pressure’ against different parts of the bed net should be positively correlated with the potential for mosquitoes to enter the net in those areas when holes are present. These experiments provide new information and insights on mosquito-ITN interactions by examining the effects of the following on mosquito pressure: 1) presence/absence of a human in the bed net; 2) different human subjects in the bed net; 3) mosquito species (*An. gambiae* and *Anopheles albimanus*) released around the bed net; 4) insecticide treated *vs* untreated nets; and, 5) ambient conditions (i.e., ‘cool, dry’ conditions *vs* ‘warm, humid’ conditions).

## Methods

### Source colonies

Mosquitoes for experiments were drawn from stock colonies of *An. gambiae s.s.* (G3 strain) maintained in the Malaria Branch at the Centers for Disease Control and Prevention (CDC) and of *An. albimanus s.s.* (MRA-126, MR4, STECLA^a^) maintained by the Malaria Research and Reference Resource Center (MR4). Both colonies were housed in the insectary facilities of the CDC in Atlanta, Georgia, USA. Larvae, pupae and adults of both species were maintained at 28°C on a 12 hr: 12 hr light: dark cycle with a 30-min ‘sunrise’ and ‘sunset’. Adults emerged directly into 4-L cylindrical cardboard containers (*An. gambiae*) or 4-L plastic pails (*An. albimanus*). Adults of both species were provided with carbohydrates *ad libitum* in the form of 10% corn syrup in the case of *An. gambiae* and 10% sucrose in 2% methyl paraben in the case of *An. albimanus*.

### Mosquitoes for experiments

Experiments used four to eight day-old nulliparous, not previously blood-fed females. Early in the afternoon of the day of the planned experiment, an appropriately aged container of mosquitoes was put into to a cubic 33 cm × 33 cm × 33 cm mesh containment cage (‘rearing cage’- BioQuip cat. #1468B) where its lid was removed releasing the mosquitoes into the cage. Up to 50 females were gently mouth-aspirated from the cage into each of several (maximum four) screw-top polystyrene vials (approx 3 cm wide × 8 cm tall) with screen across one end. To help ensure they were blood hungry, females were drawn from those that settled on, and tried to probe through, the containment cage’s mesh sleeve when it was placed above a dish of warm water.

### Bed nets

Experiments used rectangular 150 cm high × 180 cm long × 130 cm wide white polyester (75 denier) insecticide-treated (PermaNet 2.0® containing 55 mg/m^2^ deltamethrin) or identically made, untreated bed nets supplied by Vestergaard-Frandsen Co Ltd. Each net was supported by a 130 cm × 180 cm light metal rod frame supporting the roof and by another frame of the same dimensions supporting, and giving form to, the bottom edge. The treated nets’ insecticidal efficacy was confirmed with cone tests^b^ with *An. gambiae* performed before and after the eight-month experimental period and with *An. albimanus* at the end of the experimental period.

### Sticky squares

‘Mosquito pressure’ on the bed nets was determined by sampling mosquitoes around the bed nets with 30 cm × 30 cm ‘18×16’ fibreglass (18 mesh spaces/linear inch (7/cm) in the ‘x’ dimension by 16 mesh squares (6.3/cm) per linear inch in the ‘y’ dimension) sticky screen squares hung directly on the net with S-hooks improvised from paper clips. To prepare them, each screen square was placed on a piece of cardboard and sprayed liberally with Tangletrap® which was then was spread over the screen square with a gloved hand to ensure full coverage. The square was turned over, placed back on the cardboard and sprayed again. Finally, the square was lifted from the cardboard and any holes that had been occluded by accumulations of Tangletrap were cleared. Sticky squares were left for at least 24 hr to ‘cure’ before being used in experiments.

Two groups of 18 sticky squares each were made. One group was used in experiments with treated bed nets and the other with untreated bed nets. Sticky squares in each group were numbered 1–18 on a small paper label stapled to the square edge. The label also identified ‘front’ and ‘back’ sides of the squares. Each group of sticky squares was stored separately from the other in a stack protected top and bottom with two or three additional sticky squares. Both groups of sticky squares were retreated twice over the course of the experiments.

### Experimental set-up

**Figure 1:**
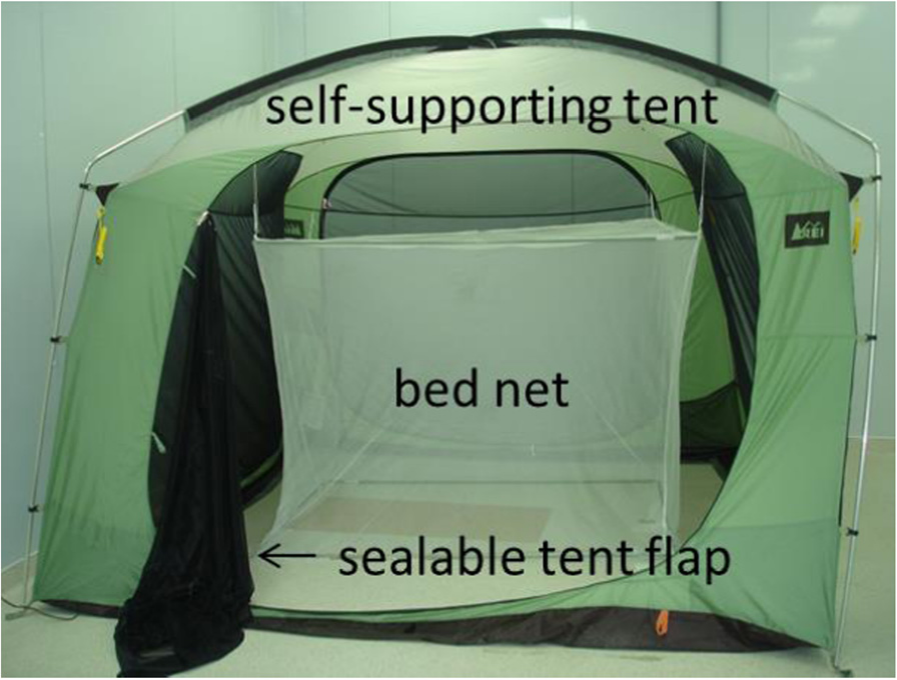
**Photograph of a tent and a bed net hung in it.** Subjects entered the tent through the near side flap which, when closed, made the tent mosquito-tight.

Each experiment was performed in one of three 3 m × 3 m × 2.1 m tall REI® screen houses (subsequently referred as the ‘tents’) (REI catalog #794-289-0018) (Figure 1). The skirt around the inside bottom of each tent was taped to the floor to prevent mosquitoes from escaping. Each tent had a single treated or untreated bed net hung in it so that the long dimension of the bed net was parallel to the ‘front’ of the tent which was defined as the face of the tent with the flap that was normally used for entry and egress.

Three tent-bed net assemblies were used, one in each of three large experimental rooms (each room approximately 10 m × 5 m × 5 m high). Two of these rooms (referred to as ‘ambient rooms’) operated under general building temperature and humidity conditions. One ambient room was reserved for experiments with treated bed nets and the other was reserved for experiments with untreated bed nets. The third room, an environmental chamber that was fully adjustable for temperature, relative humidity and lighting regime, was used for experiments with untreated bed nets. To limit the effect of the turbulence created by ventilation fans in the environmental chamber, the tent in this room was completely covered (except for a roughly 80 cm × 80 cm section of the top) with several light (0.3 mm thickness) plastic sheets.

**Figure 2:**
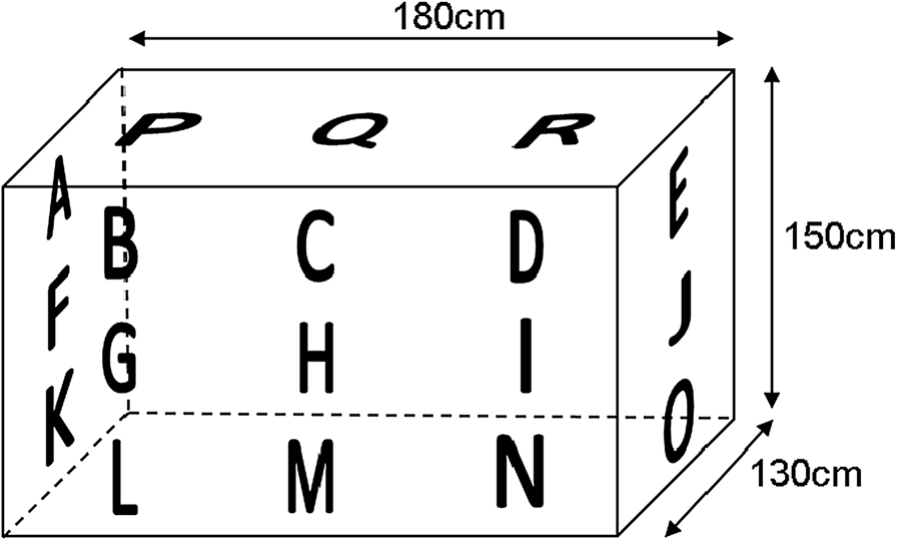
**Diagram of bed net showing approximate locations of sticky squares in the 18 areas sampled.** The back (far) side of the bed net was not sampled. Letter designations are always in the same location relative to the subject’s position in the net (also see Figure 4).

**Figure 3:**
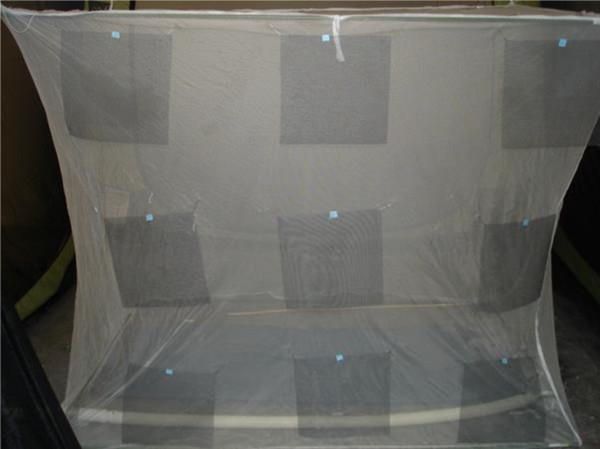
**Photograph of a bed net with sticky sampling squares hung on the side.** (Corresponding to positions B-D, G-I and L-N in Figure 2).

**Figure 4:**
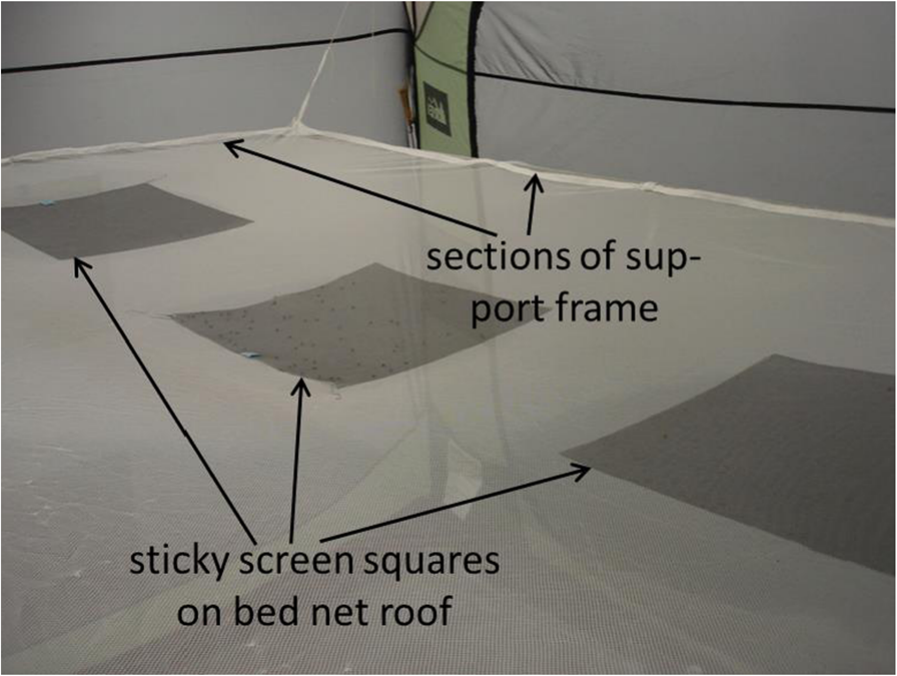
**Photograph of the roof of the bed net after an experiment with *Anopheles gambiae *showing three sticky sampling squares in place (locations P-R) and sections of the bed net support frame.** A large number of mosquitoes have been caught on the center square (location Q).

For sampling mosquitoes around bed nets, a single sticky screen square was placed in the middle of each of 18 pre-determined sampling areas on the bed net (Figure 2). There were three such locations on each end at low, middle and high levels, nine such locations on one side at low, middle and high levels (Figure 3) and three locations on the net top (roof) at positions roughly above where the prone subject’s head, mid-section and feet would be (Figure 4). Experiment-specific locations for each sticky square in the numbered set were determined from a random number table. The side of the bed net away from the tent entrance was left free of sticky squares to facilitate subject bed net entry and egress. All experiments were started in the mosquitoes’ subjective early evening.

Preliminary observations showed that *An. gambiae* were largely inactive when room lights were left on during experiments. Accordingly, all experiments with this species were done with room lights off (light levels too low to measure using Hobo® data loggers). *Anopheles albimanus* females did not appear to be affected by lighting conditions so results with room lights on and with room lights off were pooled for this species.

### Human subjects

Three volunteers were used as human subjects in experiments. Recruitment and management of volunteers were done under approved human research protocols from the CDC Institutional Review Board (Protocol 6319) and Trent University Research Ethics Board (Protocol #22550). Subject 1 was a 62-year old male, 1.75 m tall, 74.8 kg; subject 2 was a 38-year old male, 1.73 m tall, 83.9 kg; and, subject 3 was a 22-year old female, 1.58 m tall, 55.8 kg. Subjects wore no heavy perfumes or colognes during experiments and wore a basic ‘uniform’ consisting of shorts, socks and t-shirt. No special measures were taken to standardize subject factors beyond these.

### Experimental procedure

After the sticky squares had been hung on the bed net, release vials with a known number (up to 200) of mosquitoes in them were put (screw tops loosened but still in place) on the floor in the tent about 60 cm from the sticky square-free side of the bed net. The subject then zipped the tent closed, entered the bed net from the sticky square-free side and secured the bed net edges under the bottom frame leaving a small gap adjacent to the release vials. Once in position, the subject reached through the gap with a short stick to knock the vials over thus releasing the mosquitoes. The gap along bed net edge was then quickly secured from inside by the subject who then lay down in the bed net for the duration of the experiment. No one else was present in the tent or the room during experiments.

Subjects lay uncovered on a low cot or on a combination of a thin air mattress, foam mat and blanket placed directly on the concrete floor. To control for possible orientation bias, each subject was positioned in roughly half of the experiments for a given mosquito species and set of conditions with his/her head at the left and feet to the right in the bed net and in the other half of the experiments in the opposite direction. Sampling location designations (locations A to R) were adjusted accordingly. While they were free to shift position slightly during experiments to be comfortable, subjects were asked to try to be still as much as possible and lie on their backs looking upward with their hands at their waists or sides and legs extended. In experiments done in darkness, subjects were equipped with a flashlight which they used from time-to-time make brief observations of mosquito activities on the outside of the bed net and to get in and out of the bed net at the beginning and end of the experiment.

After two hours, the subject exited the bed net and the number of mosquitoes on each sticky square was counted. Control experiments were performed according to the same protocol except that there was no subject in the bed net.

### Ambient conditions and experimental treatments

*Cool and dry* – this term is used for the conditions that applied to all experiments done in ambient rooms. Over the period of the study, actual conditions in these rooms ranged from 22-24°C and 35-55% relative humidity (RH) though, during any given experiment, conditions varied by no more than approximately plus or minus 0.5°C and 5% RH.

*Warm and humid* – these conditions could only be achieved in the environmental chamber. For experiments done in these conditions, temperature was set to 28°C and RH to 80%. Actual conditions varied from set values by no more than approximately plus or minus 0.5°C and 3% RH.

### Statistical analysis

The distribution of mosquito catch at each sampling location on the bed nets and in total on bed nets was non-normal. Therefore, for statistical purposes, total catch data (adjusted to an equivalent release total of 200 mosquitoes) for each treatment (expressed as mean total catch for descriptive purposes in the Results) were ranked and compared by non-parametric Wilcoxon tests between groups. For each mosquito species, specific comparisons were made between subject 1 in the untreated, cool, dry condition and all other conditions. A direct comparison of species was also done for subject 1. For illustration and discussion purposes, the catch on a given sticky square is assumed to be representative for the entire location surrounding it.

The distributions of mosquitoes on bed nets for each treatment were analysed using hierarchical cluster (HC) analysis. In this analysis, each of the 18 sampling locations for a given set of experimental conditions is initially regarded as a discreet cluster. The HC algorithm then creates a hierarchy of clusters in a step-by-step process that merges clusters according to their Euclidean distances from each other. This occurs sequentially until a single cluster is formed. In the interpretation of the HC analysis, the number of clusters considered valid for each set of experimental conditions was limited to, in most cases, the first three that were resolved through this process. In three cases, four clusters were resolved. Note that in the Results, clusters are listed in order of descending mean catch.

## Results

**Figure 5:**
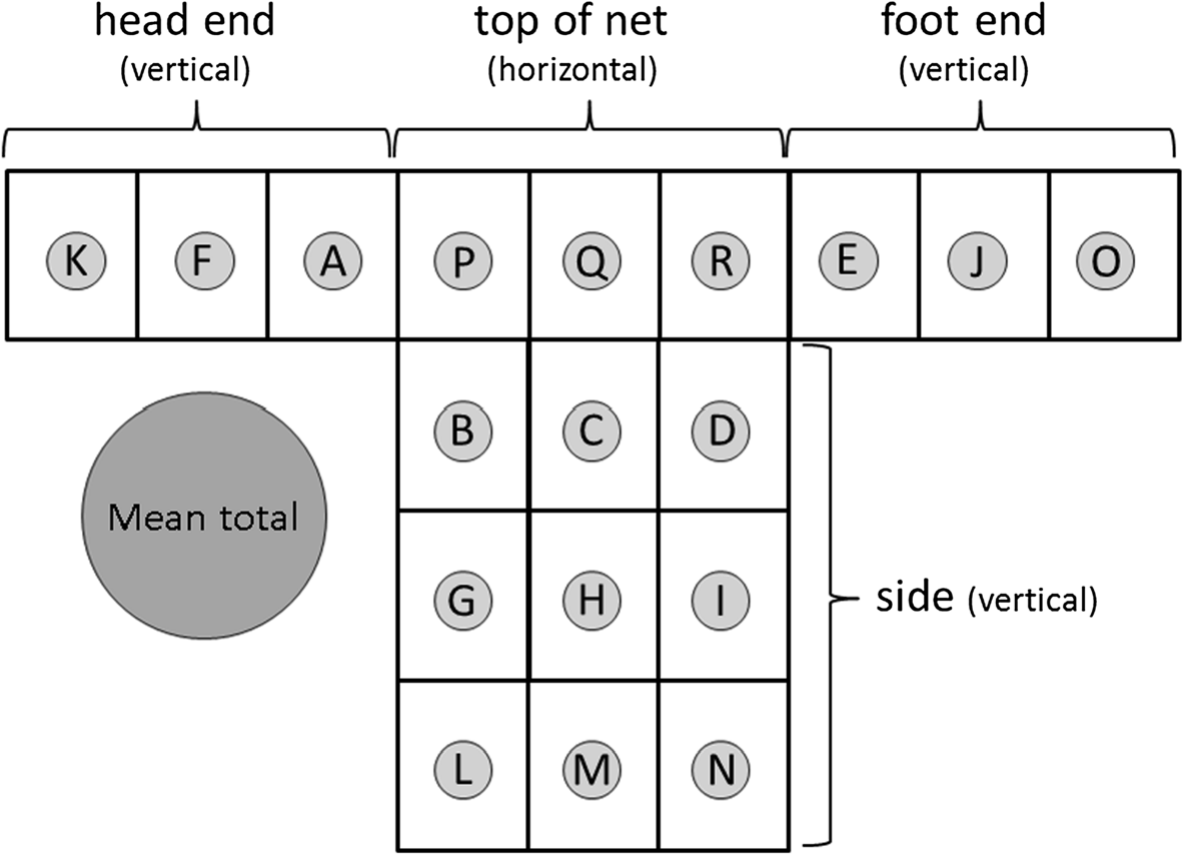
**Template diagram for catch distribution figures (Figures **6**, **7**, **8**, **9**, **10**, **11**, **12**, **13**, **14**, **15** and **16**).** Sampled part of bed net is depicted as folded out and viewed from above (non-sampled side panel not shown). For each diagram, the number in the circle at each sampling site on the bed net (A-R) is mean catch at that location; the circle area represents proportion caught at that location. The area of the largest circle on the left of the diagram is proportional to total mean catch for the bed net under the conditions given. Scale of large circles is the same in all diagrams.

In total, 71 experiments were performed, 45 with *An. gambiae* and 26 with *An. albimanus* (Table 1). Results for each species and set of experimental conditions are presented in Figures in a subject orientation-corrected form in which the letters A to R designate the 18 sampling locations on the bed net. See Figure 5 for an explanation of results diagram format and interpretation.

**Table 1:** **Number of experiments done with treated and untreated nets in cool, dry and warm, humid ambient conditions with *Anopheles gambiae*/*Anopheles albimanus *** **using subjects 1–3 and no host (control) in bed net**

**Bed net type**	**Ambient conditions**	**Subject**			
		**Control**	**1**	**2**	**3**
Untreated	Cool, dry	6/4	13/8	7/5	5/-
Treated	Cool, dry	4/2	5/4	−/−	−/−
Untreated	Warm, humid	−/−	5/3	−/−	−/−

### Effect of a host in the net

**Figure 6:**
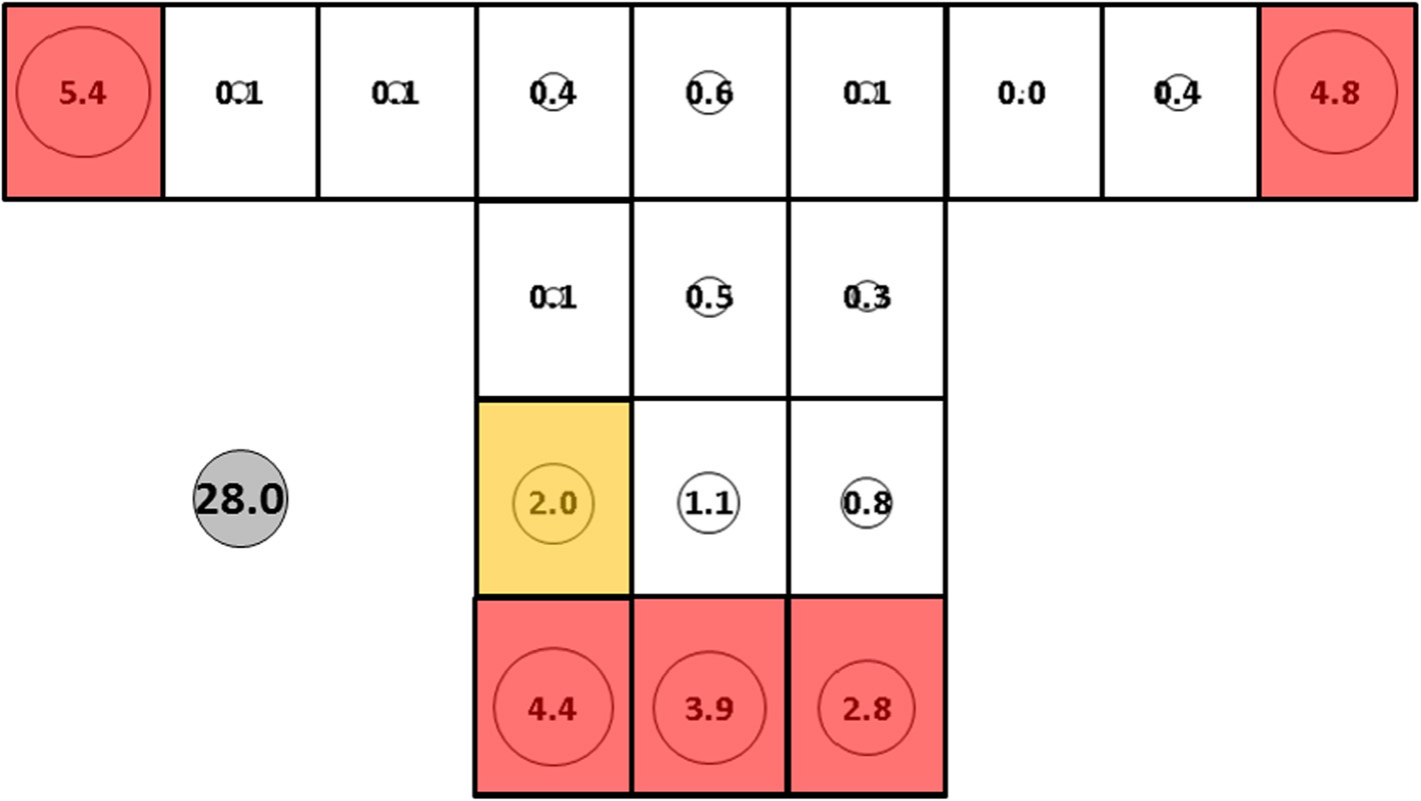
***Anopheles gambiae*, control (no subject), under dry, cool conditions in untreated bed net.** See Figure 5 for an explanation of general diagram layout. Cluster colour code: red – greatest mosquito pressure, orange – second greatest mosquito pressure, green (if present) – third greatest mosquito pressure, white – lowest mosquito pressure.

**Figure 7:**
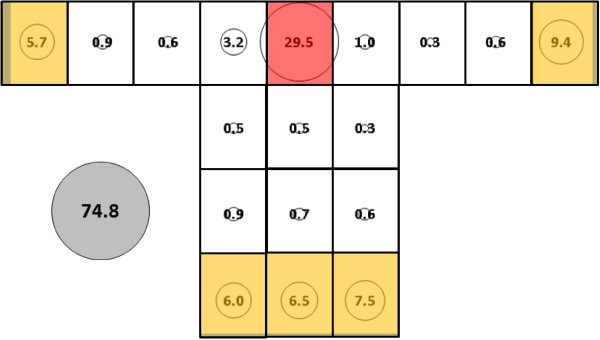
***Anopheles gambiae*, subject 1, under dry, cool conditions in untreated bed net.** See Figure 5 for an explanation of general diagram layout. Cluster colour code: red – greatest mosquito pressure, orange – second greatest mosquito pressure, green (if present) – third greatest mosquito pressure, white – lowest mosquito pressure.

Mean total control (no subject in the net) catch for *An. gambiae* was 28.0 (SE = 14.6) (Figure 6) while total mean catch with subject 1 in the net was significantly higher at 74.8 (SE = 11.3) (Figure 7) (p < 0.0002). Presence of the subject resulted in at least small catch increases at all sampling locations on the bed net with the exception of locations B, C, and G-I at mid and upper levels of the side panel. Subject presence also resulted in large numeric and proportional increases at location Q on the net roof. Analysis resolved three clusters of sampling locations on the control net: 1) all locations along the bottom of the net (K-O); 2) location G; and, 3) all other locations including those on the net roof. For the host-present condition, the three clusters were: 1) location Q; 2) locations K-O; and, 3) all other locations.

**Figure 8:**
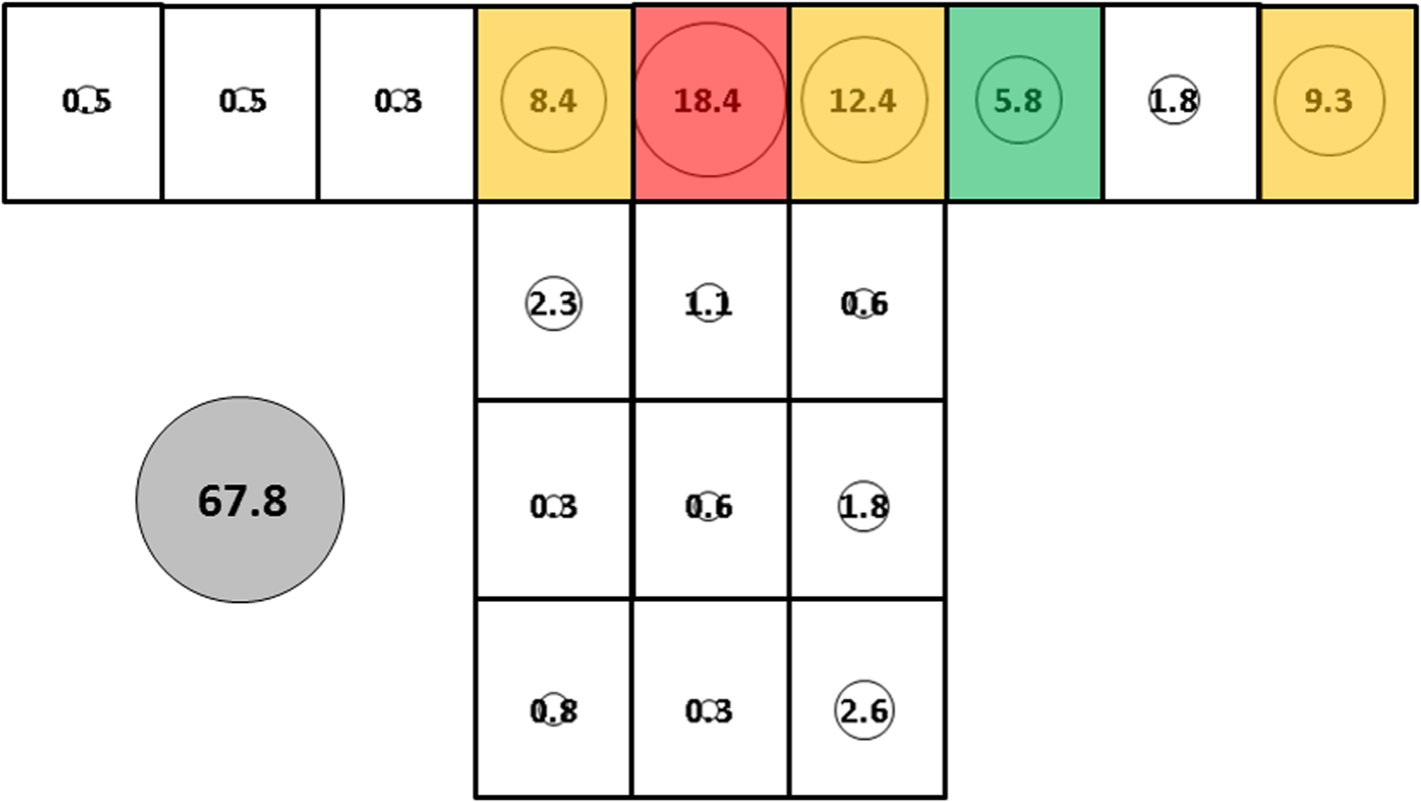
***Anopheles albimanus*, control (no subject) under cool, dry conditions in untreated bed net.** See Figure 5 for an explanation of general diagram layout. Cluster colour code: red – greatest mosquito pressure, orange – second greatest mosquito pressure, green (if present) – third greatest mosquito pressure, white – lowest mosquito pressure.

**Figure 9:**
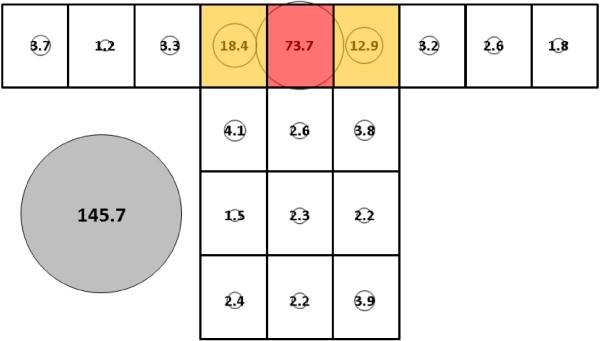
***Anopheles albimanus*, subject 1, under cool, dry conditions in untreated bed net.** See Figure 5 for an explanation of general diagram layout. Cluster colour code: red – greatest mosquito pressure, orange – second greatest mosquito pressure, green (if present) – third greatest mosquito pressure, white – lowest mosquito pressure.

For *An. albimanus*, total mean catch was 67.8 (SE = 30.7) (Figure 8) for the control bed nets, which was significantly lower than 145.7 (SE = 14.2) (Figure 9) for the subject 1-occupied bed net (p < 0.0063). While subject presence resulted in significantly greater total mean catch for *An. albimanus*, it did not result in as profound a redistribution to the top of the net as was observed with *An. gambiae.* For the no-host *An. albimanus* condition, four clusters were resolved: 1) Q; 2) O, P and R; 3) E; and, 4) all other locations (i.e., all side and end locations except E and O) while three were resolved for the host-present condition: 1) Q; 2) P and R; and, 3) all other locations (i.e., all side and end positions).

### Effect of host individual

**Figure 10:**
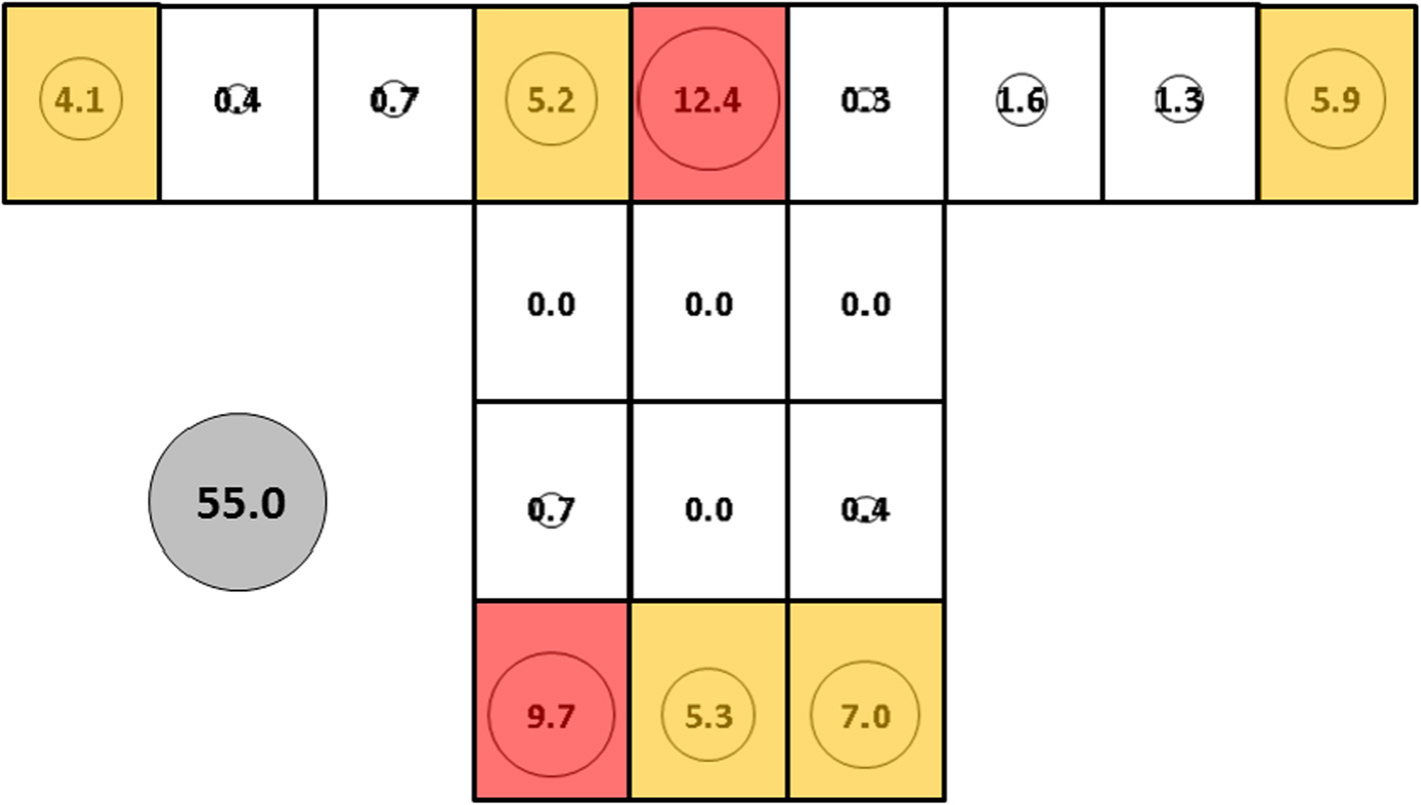
***Anopheles gambiae*, subject 2, under dry, cool conditions in untreated bed net.** See Figure 5 for an explanation of general diagram layout. Cluster colour code: red – greatest mosquito pressure, orange – second greatest mosquito pressure, green (if present) – third greatest mosquito pressure, white – lowest mosquito pressure.

**Figure 11:**
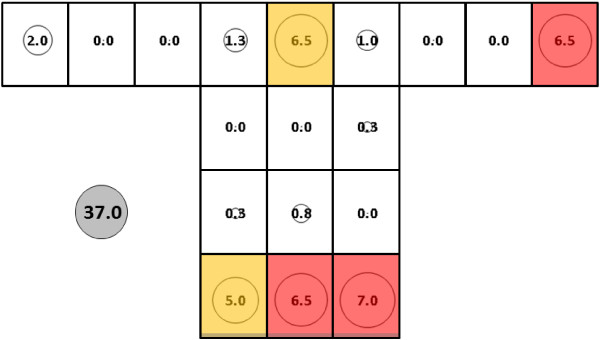
***Anopheles gambiae*, subject 3, under dry, cool conditions in untreated bed net.** See Figure 5 for an explanation of general diagram layout. Cluster colour code: red – greatest mosquito pressure, orange – second greatest mosquito pressure, green (if present) – third greatest mosquito pressure, white – lowest mosquito pressure.

In experiments with *An. gambiae*, total mean catch for subject 2 (55.0, SE = 10.9, Figure 10) and for subject 3 (37.3, SE = 12.0, Figure 11) did not differ significantly from each other or from the subject 1 catch (Figure 9). Cluster analyses for all subjects resolved three groupings with the mid and upper level locations on the side making up the least productive cluster (cluster 3) in all cases. Details of clusters 1 and 2 make-up differed somewhat between subjects as mean catch decreased from subject 1 to subject 2 and then to subject 3. For subject 2, cluster 1 consists of sampling locations L and Q while for subject 3, cluster 1 consists of locations M, N and O along the bottom of the net.

**Figure 12:**
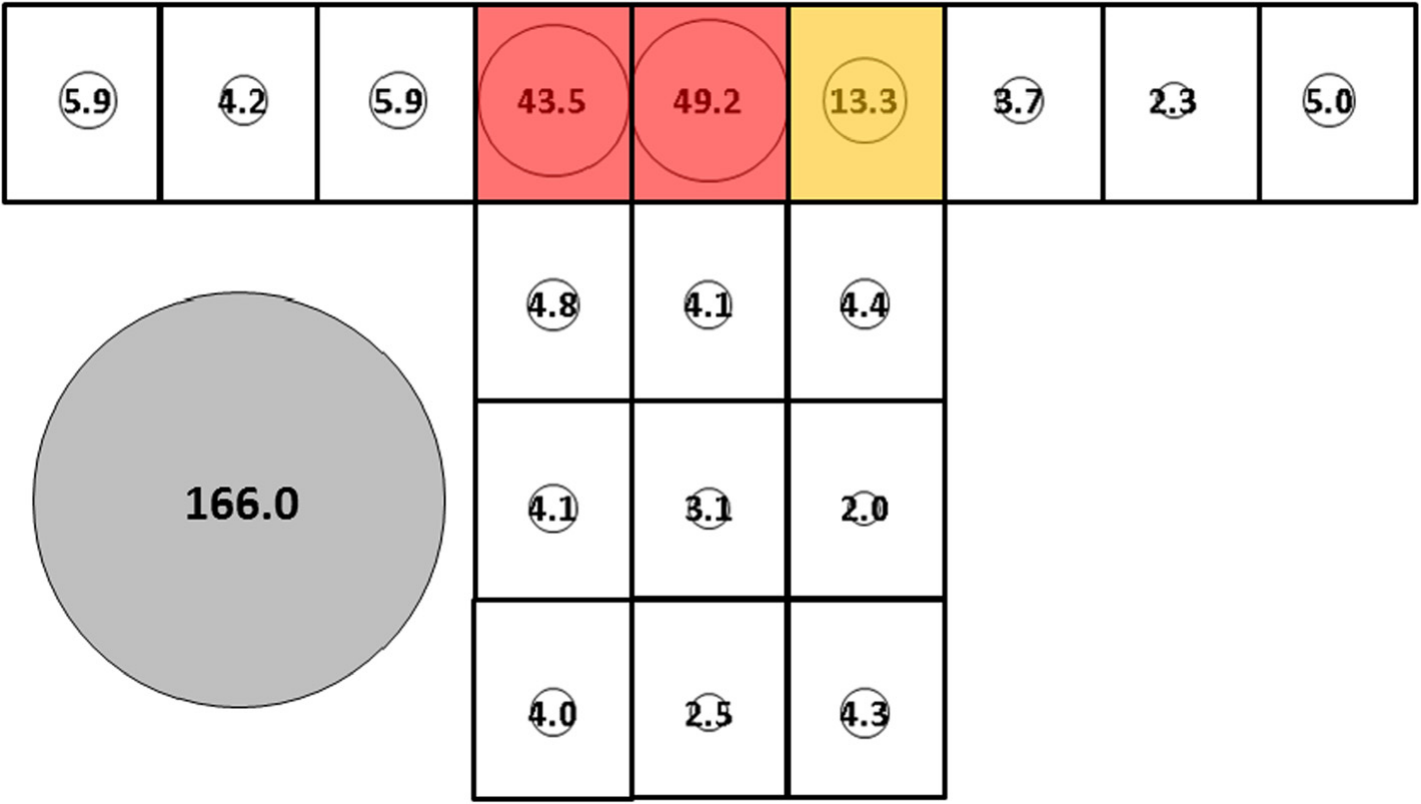
***Anopheles albimanus*, subject 2, under cool, dry conditions in untreated bed net.** See Figure 5 for an explanation of general diagram layout. Cluster colour code: red – greatest mosquito pressure, orange – second greatest mosquito pressure, green (if present) – third greatest mosquito pressure, white – lowest mosquito pressure.

Mean total catch for *An. albimanus* for subject 2 (166.0, SE = 14.2, Figure 12) did not differ significantly from subject 1 mean total catch (145.7, SE = 14.2, Figure 9). Over 90% of mosquitoes were caught on the roof of the bed net in both cases. Mosquito catch on the subject 2-occupied net resolved into three clusters: 1) P and Q; 2) R; and, 3) A-O. The sole difference between the subjects was that location P grouped with location R for subject 1 but with location Q for subject 2.

### Mosquito species effect

Subject 1 total mean catch for *An. gambiae* was significantly lower than for *An. albimanus* for (74.8, SE = 11.3 *vs* 145.7, SE = 14.2, p < 0.001). See 'Effect of a host in the net' (above) and Figures 7 and 9 for descriptions of the mosquito catch and distributions for each species.

### Cool, dry *vs* warm, humid effect

**Figure 13:**
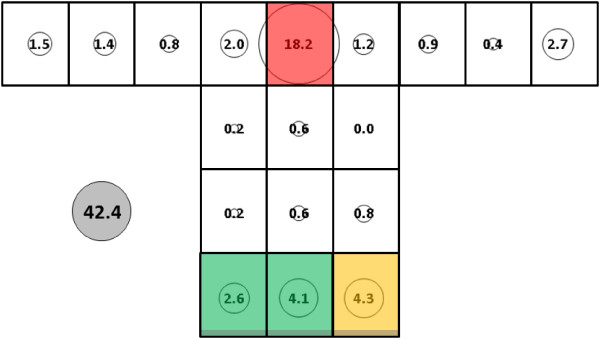
***Anopheles gambiae*, subject 1, under warm, humid conditions in untreated bed net.** See Figure 5 for an explanation of general diagram layout. Cluster colour code: red – greatest mosquito pressure, orange – second greatest mosquito pressure, green (if present) – third greatest mosquito pressure, white – lowest mosquito pressure.

With subject 1 in the net, warm, humid conditions did not result in significantly different mean total catches for *An. gambiae* (Figure 13, 42.4, SE = 6.9) when compared to the mean total catches for subject 1 under cool, dry conditions (74.8, SE = 11.3). Analysis of the catches in warm, humid conditions resolved four clusters: 1) Q; 2) N; 3) L and M; and, 4) all other locations. This pattern is similar to the pattern for subject 1 under cool, dry conditions except that locations K and O in the warm, humid condition were part of the least productive fourth cluster.

**Figure 14:**
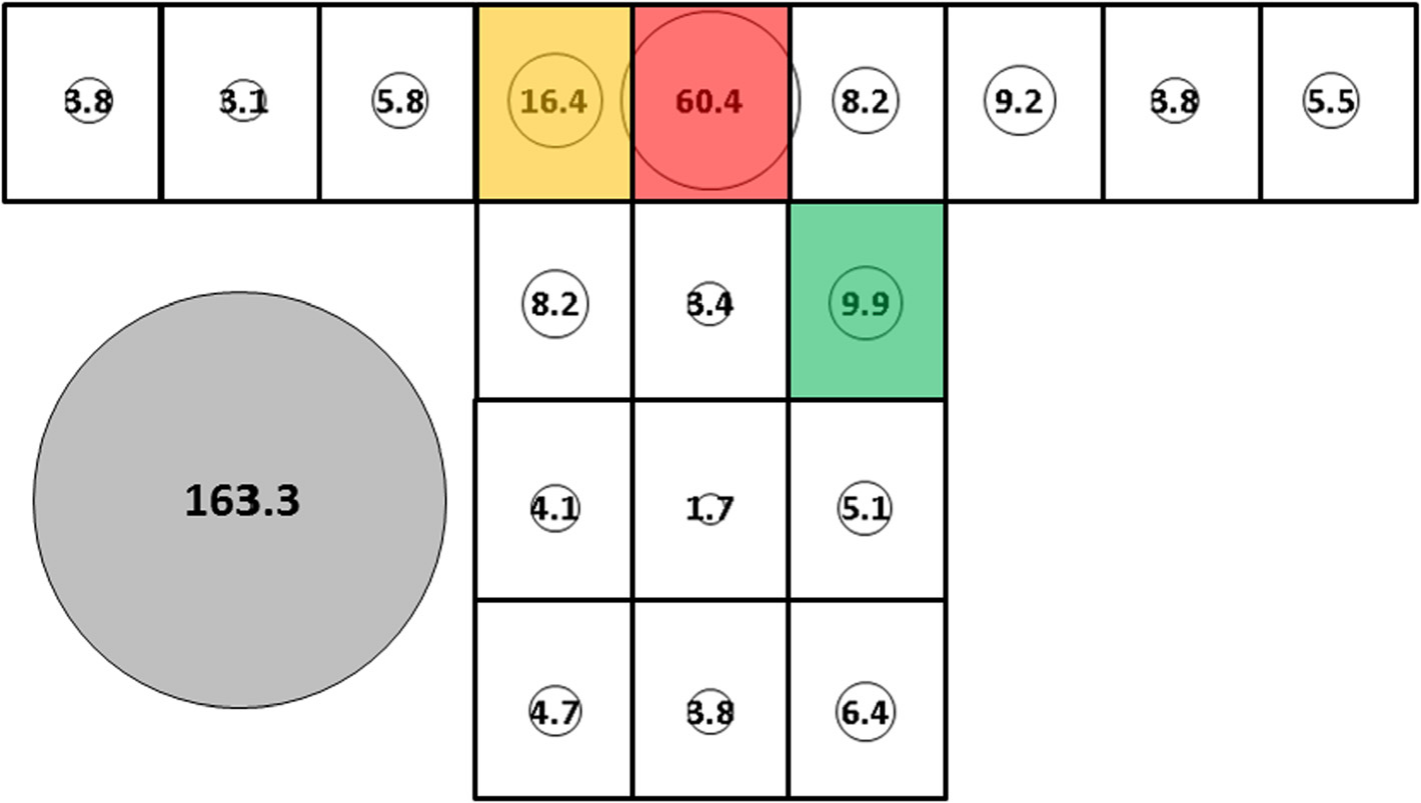
***Anopheles albimanus*, subject 1, under warm, humid conditions in untreated bed net.** See Figure 5 for an explanation of general diagram layout. Cluster colour code: red – greatest mosquito pressure, orange – second greatest mosquito pressure, green (if present) – third greatest mosquito pressure, white – lowest mosquito pressure.

**Figure 15:**
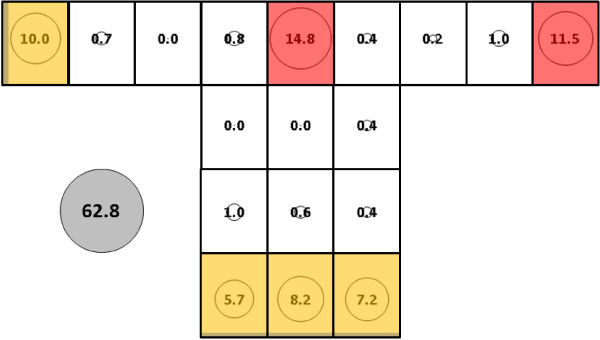
***Anopheles gambiae*, subject 1, under cool, dry conditions in treated bed net.** See Figure 5 for an explanation of general diagram layout. Cluster colour code: red – greatest mosquito pressure, orange – second greatest mosquito pressure, green (if present) – third greatest mosquito pressure, white – lowest mosquito pressure.

Compared to subject 1 under cool dry conditions, warm humid conditions produced significantly greater mean total catches of *An. albimanus* (Figure 14, 163.3, SE = 19.0 *vs* Figure 15, 62.8, SE = 16.0, p < 0.02). The additional catch in warm, humid conditions was mainly at several locations on the sides and ends of the bed net (in particular, locations D, E and O), which more than made up for smaller numbers on the roof. Cluster analysis resolved four groupings in this case: 1) Q; 2) P; 3) D; and, 4) all other locations.

### Treated *vs* untreated net effect

Mean total catch of *An. gambiae* on the treated bed net occupied by subject 1 (Figure 15, 62.8, SE = 16.0) did not differ significantly from the untreated net occupied by subject 1 (Figure 7, = 74.8, SE = 11.3). Clustering was also similar for both conditions though, for the treated net, location O joined location Q as part of cluster 1.

**Figure 16:**
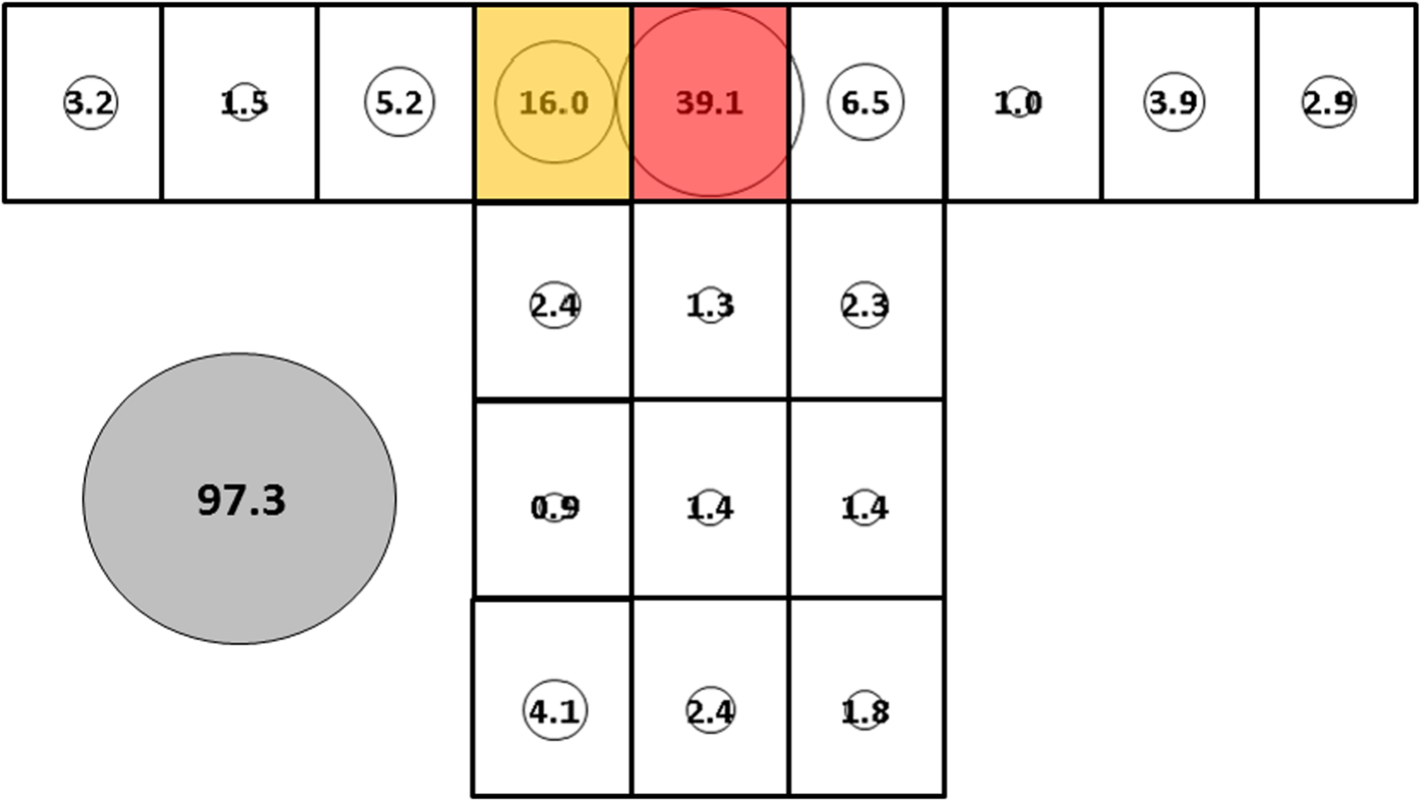
***Anopheles albimanus*, subject 1, under cool, dry conditions in treated bed net.** See Figure 5 for an explanation of general diagram layout. Cluster colour code: red – greatest mosquito pressure, orange – second greatest mosquito pressure, green (if present) – third greatest mosquito pressure, white – lowest mosquito pressure.

Mean total catch for subject 1 of *An. albimanus* on treated nets was significantly lower (Figure 16, 97.3, SE = 9.5 *vs* Figure 14, 163.3, SE = 19.0, p < 0.05) than on its untreated counterpart. Clustering on the treated bed net was similar to the untreated net though for the treated net all roof locations caught a smaller proportion of the total and location R moved from cluster 2 (which consisted of locations R and P on the untreated net) to cluster 3.

## Discussion

### Comments on experimental design

The sticky square method, while providing less spatial resolution than the method used by Lynd and McCall [23] in which the entire bed net was sticky, was chosen for these experiments because it provided a way to sample from several areas of the net while leaving most of it uncovered. Approximately 82% of the sampled faces of the bed net (roof, both ends and one side), and 86% of the entire net, was free of sticky squares. This was especially important for showing how mosquitoes react to treated nets. In direct observation, neither mosquito species appeared to avoid the sticky squares or to be drawn to them. Sticky squares were not perfectly efficient. Mosquitoes were often observed to contact sticky squares and then fly away. Therefore, numbers caught at each sticky square are interpreted as representative of the relative amount of time mosquitoes flew against the net in each area (for present purposes it is assumed that sticky square catch in a given area is representative of the entire area surrounding it although this is probably an unrealistic simplification) and not as where the mosquito first contacted the net. It is assumed that that the number of mosquitoes entering the bed net through a given size hole in a given area would be proportional to this ‘mosquito pressure’ in that area (see below for further discussion of this point).

As designed, these experiments sampled all locations at the same time, i.e., competitively. This means that locations that caught more mosquitoes (e.g., location Q) will have made a certain number of mosquitoes unavailable to be caught at other locations later. Thus, the experimental design is biased somewhat to overestimate mosquito pressure at, for instance, location Q and underestimate it at other less favoured locations. This bias is likely quite small however and could be addressed through experiments in which pressure at various locations on the net is sampled using a non-depleting method such as video recording.

Another consequence of the experimental design is that one side of the net was not sampled. This was done so that the experimenter could enter and exit the net without disturbing sticky squares. Had both sides been sampled, number caught at the other locations would likely have been reduced somewhat but, since there was an excess of mosquitoes released, this effect would probably have been small. Elsewhere in the Discussion, it is assumed that mosquito pressure on the unsampled side is a mirror image of that on the sampled side. This should reflect the situation around a real-life bed net more accurately than would compensated mosquito pressures because in real-life, mosquitoes would be flying freely around the bed net without the risk of being caught on a sticky square.

### Behavioural interpretations

#### Effect of a host in the net and species effect

In the ‘empty-net’ condition, almost all *An. gambiae* were caught on the lowest sampling locations suggesting that, after release, mosquitoes stayed largely at floor level during the experiment. This would explain the very few (none in some experiments) caught at higher locations and is consistent with what is known of this species’ behaviour, which is that it flies close to the ground much of the time. Snow [24] found that 80% of unbaited suction trap catches of *An. gambiae s.l.* dispersing from an area of rice paddies in The Gambia were within 1 m of the ground.

The presence of a human subject in the bed net significantly increased the total pressure from both species in comparison to the empty-net condition. The overall relative catch increase was approximately 2.7× for *An. gambiae* and occurred at most lower level sampling locations (1.5-3× increases) and at locations on the roof, especially at location Q (30× increase) but not at locations at the mid and upper levels of the side and head end of the net. The large pressure increase on the net roof is consistent with Lynd and McCall [23] though they also found larger increases at mid and high levels on the side near the head end of the occupied bed net.

Observations with the subject present are consistent with increased overall activity levels resulting from host-originating elevated levels of CO_2,_ a known activator of host-seeking behaviour in *An. gambiae*[18,19]. When host seeking, *An. gambiae,* readily flies upward to heights of a couple of metres or more when it encounters vertical barriers such as outside walls of houses [25]. When a subject was present thus activating host seeking responses, the same upward flight response may have occurred when *An. gambiae* females encountered the sides and ends of the bed net or the inside walls of the tent. This could account for the continual presence of *An. gambiae* flying high in the tent during experiments.

**Figure 17:**
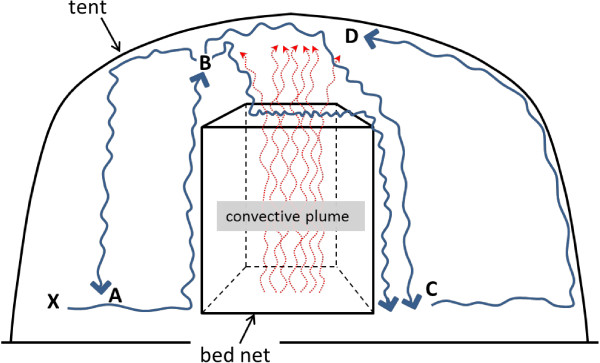
**Hypothesized pattern of mosquito movement in tent containing an occupied bed net (human subject not shown).** Red arrows represent rising convective plume from subject. ‘X’ represents mosquito experimental release point. See text for explanations of the blue arrows and other letters.

The combination of behaviour and the tent environment provides a basis for the proposed model of *An. gambiae* circulation in the tent illustrated in Figure 17. In this model, mosquitoes released at point X fan out at a low level (A in the diagram) until they encounter a vertical surface such as the side of the bed net or the tent. They then fly upward many getting up high in the tent (at B in the diagram) where there are various possibilities. One possibility is that, once away from the vertical surface, some drop back down to floor level (A or C) where their natural tendencies may cause them to fly upwards again when they re-encounter the bed net or tent sides (B and D). Another possibility is that they come into contact with the convective plume above the bed net and follow it downward. This is supported by Dekker *et al.*[22] who showed that *An. gambiae s.s.* move down presumed host-originating convection currents looking for a place to bite. In the context of these experiments, mosquitoes would have moved down the convective plume until being stopped by the net roof where they were arrested by the warmth, moisture and, possibly, breath and skin volatiles in the rising plume. This is consistent with the large catches of *An. gambiae* on the roof, especially at location Q and is supported by the periodic direct observations made from inside the bed net during experiments (with the aid of a flashlight) which often revealed some mosquitoes in low skimming flight over the net roof, others ‘bouncing’ along the roof and others walking slowly on the net roof sometimes probing through the mesh. In this model, some of these mosquitoes would eventually slip or fly over the edge of the roof where they would lose contact with the plume and then drop to the floor. This is supported by the very low numbers caught on mid and upper level locations on the sides and ends of the bed net and by the fact that direct visual observation from inside the bed net revealed very few mosquitoes in contact with, or close to, the net sides at those levels. While this model could explain much of what was observed, it does not address the reasons for the concentration of roof catches at the middle location (Q) of net roof, as opposed to locations R and P or the specifics of roof distribution already demonstrated in previous work [23]. This may relate to where on the net roof key features of the rising plume were strongest though currently there is no data that addresses this. In a real life situation where mosquitoes often enter houses at the eaves, this same pattern of movement may apply. Once having entered the house through the eaves, host seeking *An. gambiae* may slowly drop back down to floor level since this is their preferred flight level but then fly upward again upon encountering walls or the sides of bed nets with sleepers inside.

Given the strong effect of foot odours on *An. gambiae* biting site selection [26,27], it was surprising not to see higher pressures at locations near the feet, especially location O. Location O accounted for more *An. gambiae* than the corresponding low level location at the head of the bed net (location K) but these differences were small and did not differentiate from one another in cluster analyses. The apparent absence of a ‘foot effect’ may illustrate the difference between pre-contact close range orientation to the subject, which may be driven largely by the convective plume, and landing or biting site selection which probably occurs at very close range. It is also possible that subjects’ socks and feet were too clean to elicit the foot odour effect this species is known for.

The empty-net condition for *An. albimanus* contrasts with the same condition for *An. gambiae* most noticeably in producing much larger absolute and proportional pressures on the roof of the net. This suggests that *An. albimanus* is more generally active than *An. gambiae* and that it tends to fly higher than *An. gambiae* in the absence of host stimuli. The increased overall catch with the subject present for *An. albimanus* is consistent with generally increased flight activity elicited by CO_2_ or other subject-related cues serving as activating stimuli. The subject-present condition for *An. albimanus* produced a smaller redistribution effect than for *An. gambiae.* Despite this*,* the reduced pressures at locations E and O suggest that the subjects’ presence elicited a strong response to the top of the bed net.

In terms of the circulation model presented for *An. gambiae* inside the tent (Figure 17), these results suggest that *An. albimanus* is more free-flying and less bound to flying close to the ground than *An. gambiae.* This could account for the quite small catches of *An. albimanus* at all side and end locations including low-level locations with the subject present. The results also suggest that *An. albimanus* responds to the subject’s convective plume in a manner similar to *An. gambiae*, a conclusion that is consistent with previous work on how host factors, including heat and moisture, affect close range host orientation in *An. albimanus*[28].

### Effect of host individual

No significant differences were found between total mean catch for the three subjects tested with *An. gambiae* or for the two tested with *An. albimanus*. It is not surprising that total mean catch did not differ significantly between subjects 1 and 2 for either species since these subjects were both male and similar in height and weight. It is somewhat surprising, however, that total mean catch for subject 3, the smallest and only female subject in *An. gambiae* experiments, though numerically lower than for subjects 1 and 2, did not differ from these statistically. This may simply be an effect of catch variability and a small number of replicates since host attractiveness is known to be affected by host size [21].

While total mean catch did not differ significantly among subjects, cluster analysis showed that, for *An. gambiae*, as total mean catch decreased, mosquito pressure patterns on the bed net shifted from being greatest on the net roof (subjects 1 and 2) to being greatest on the lower level locations (subject 3). Subject 3′s catches at lower level locations were numerically similar to those for the other subjects (except at location K where they were somewhat lower) meaning that the catch deficit was mainly on roof locations. If the proposed model for how this species circulates in the tent in the subject-present condition (see section ‘Effect of a host in the net and species effect’ and Figure 17) is correct, this could mean that subject 3 elicited similar levels of activation (due to CO_2_) but weaker attraction/arrestment/entrainment on the roof due a less intense convective plume.

Although *An. albimanus* total mean catch did not differ between subjects, cluster analysis indicates that, for subject 2, proportional mosquito catch on the roof was higher at location P (above the head) and lower at location Q (above the waist) than for subject 1. This may relate to details of structure of the respective convective plumes produced by each subject. Despite this difference, the overall pattern for this species for the two subjects tested is similar given that, for both, well over half of the mosquitoes were caught at locations on the net roof.

### Cool, dry *vs* warm, humid effect

Experiments under ‘warm, humid’ conditions were done to investigate whether the responses seen in ‘cool, dry’ experiments might differ from what could be expected in the warmer more humid conditions encountered in these species’ indigenous regions. Hypothetically, higher ambient temperature and humidity could reduce the effect of the convective plume signal because the signal would contrast less with the general environment in the tent. If so, there should be reduced catches on the roof in the warm, humid condition.

Experimental results provide no support for this hypothesis for *An. gambiae.* The mean total catch of *An. gambiae* was greater under cool, dry conditions than under warm, humid conditions but not statistically so. More importantly, mosquito pressure on the net roof (location Q), where differences would be expected if the ability of the plume to influence the mosquitoes’ behaviour had been affected, was virtually identical between treatments. Perhaps of interest in this comparison is the fact that in the warm, humid condition, the low level head and foot locations (locations K and O, respectively) did not group with the low level side locations (K-N) as they did in the cool, dry condition; rather, they grouped with the rest of the side and end locations in the cluster with lowest catches This may reflect an overall tendency for this species to fly somewhat higher in warmer conditions.

Total mean catch of *An. albimanus* was significantly greater in the warm, humid condition than in the cool, dry condition with almost all sampling locations catching more mosquitoes. The greater overall catch in warm humid conditions may reflect a temperature effect with higher temperatures eliciting higher overall activity levels. While the roof locations group somewhat differently between conditions, by far the greatest mosquito pressure is at roof locations suggesting that the convective plume effect is similar in both situations. The large proportion caught at location D in the warm, humid condition appears to reflect an overall higher catch at all upper side and end locations. Despite efforts made to eliminate air currents from the tents, these differences from the cool, dry clustering pattern may be the result of residual air movement in the environmentally controlled room getting into the tent and ‘smearing’ the plume somewhat.

### Treated net effect

The misleadingly named ‘excito-repellent’ effect [13,29], which is more correctly referred to as the ‘locomotive stimulant’ effect [30], is often cited as an important protective quality of treated bed nets since it appears to reduce mosquito entry rates into damaged bed nets [3] and to drive mosquitoes out of houses or prevent them from entering in the first place [3,31]. It was hypothesized that this apparently highly disruptive behavioural effect, combined with the insecticidal effect, would (in comparison to its untreated counterparts) 1) reduce catch and, 2) change the distribution patterns of mosquitoes on an occupied treated bed net. The insecticidal potency of the treated bed net was confirmed by cone test bioassays^b^ and by the fact that by the end of the first hour of the two-hour experiments, very few mosquitoes of either species could be observed still flying in the tents. Despite the fact that mosquitoes were available to be caught for a shorter time in treated net experiments, the first part of this hypothesis is only weakly supported since mean total catches for these experiments were either not significantly different (*An. gambiae*) or only barely significantly lower (*An. albimanus*) than mean total catches for experiments with untreated nets. The results also do not support the second part of the hypothesis since pressure patterns for treated and untreated nets for each species were similar to one another differing no more than pressure patterns among subjects. While the locomotive stimulant effect of pyrethroid insecticides (including deltamethrin) are well-supported in the literature, several studies have demonstrated that it can be overcome or delayed by host stimuli such as those in the convective plume that mosquitoes in these experiments would have been exposed to on the net roof [13,29,32,33].

### Implications for assessment of bed net physical integrity

The WHO-approved method for evaluating physical condition of bed nets uses the Proportionate Hole Index (pHI) [34] method in which holes in the bed net are counted and the counts, weighted in terms of the size class the holes fall into, but not in terms of their locations on the bed net, are totaled to yield each bed net’s pHI value. This method tacitly assumes that mosquito pressure is the same on all parts of the bed net which this work and others’ [23] show not to be the case. Assuming that mosquito pressure at a given location on the bed net is proportional to mosquito entry rate when there are holes at that location, for the species studied here, a hole of a given size in the middle of the roof of the bed net would let in many more mosquitoes than the same size hole at the mid-level on the side of the bed net. To further complicate the picture, the pHI does not consider mosquito species. These results show that there is an interaction between mosquito species and mosquito pressure pattern such that, for instance, a hole of a given size low on the net should admit relatively more *An. gambiae* than *An. albimanus* while the same hole in the mid-level on the side would admit relatively more *An. albimanus* than *An. gambiae*.

**Figure 18:**
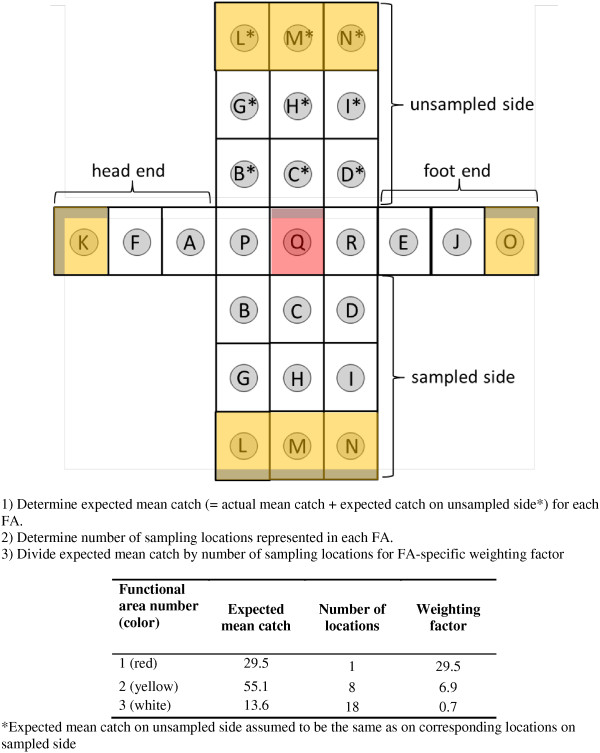
**Method for calculating functional area (FA) weighting factors using *Anopheles gambiae *results (Figure **7**).** Bed net is depicted diagrammatically as in Figure 5 but including unsampled side panel with (starred) letter designations ‘reflected’ from sampled side panel. Roof panel (squares P-R) not labelled to reduce clutter.

A behaviourally informed assessment of bed net vulnerability would weight hole importance in terms of where the holes are on the net (in addition to their size) and would take species into account. The number of areas on the bed net that would have to be differentiated for this purpose would be those that cluster analyses show are under different mosquito pressures. These are referred to as ‘functional areas’ (FAs) in the following discussion. Further data collection is needed to define the FAs on the bed net for different mosquito species but, for discussion purposes, an initial estimate of these for *An. gambiae* and *An. albimanus* can be made based on data presented here. For instance, cluster analysis of *An. gambiae* subject 1 results under cool, dry conditions in the untreated net indicates three groupings in terms of mosquito pressure (Figure 7). These are the mid-section of the roof (location Q) forming FA 1, the bottom third of the net (locations K-O and their counterparts on the non-sampled side of the net) forming FA 2, and all other locations on the net (locations A-J and their counterparts on the non-sampled side of the net, plus locations P and R) forming FA 3 (Figure 18). Using the method described in Figure 18 results in FAs 1 to 3 with weighting factors (WFs) equal to 29.5, 6.9 and 0.7, respectively. The same method using *An. albimanus* comparator data (Figure 9) also yields three FAs: FA 1 (the mid-section of the roof - location Q) with a WF = 73.7), FA 2 (both end sections of the roof - locations P and R) with a WF = 15.7 and FA 3 (the rest of the net -(locations B-D, G-I, L-N and their counterparts on the non-sampled side of the net plus locations A, E, F, J, K and O) with a WF = 2.7.

Although some studies have used the pHI to make judgments about bed net effectiveness [16,35,36], there is no consensus, nor basis for consensus, for how information about bed net physical condition can be used to define when bed nets are no longer serviceable or can be said to have ‘failed’. While inclusion of species-specific weighting factors such as these into a modified pHI calculation should yield values that are more representative of mosquito entry risk presented, these estimates would still have to be considered *relative*, not absolute. In other words, these estimates would make it possible to say how much more or less risk a bed net (or group of bed nets) presents compared to another bed net (or group of bed nets) but they would not provide an absolute estimate of the number of mosquitoes that would enter a damaged bed net under a given set of conditions. There are several reasons for this. The reason most directly related to this research is the fact that, while it is reasonable to assume that greater mosquito pressure on a part of the bed net will translate into more mosquitoes entering the net through holes in that area, the conversion factors for this relationship are not yet known. That is, does a five times greater mosquito pressure in one FA compared to another area mean that five times more mosquitoes will enter the net through a hole in that area or is the slope of the relationship between mosquito pressure and entry rate greater than 1, less than 1, or non-linear in some fashion? Determining the answers to these and related questions is among the future aims of this project and, it is hoped, will eventually allow estimates the absolute risk represented by bed nets in different states of physical deterioration and subjected to different mosquito densities and different mosquito species.

### Implications for bed net design

Presently, only a handful of studies attempt to optimize bed net design in terms of mosquito behaviour [37,38]. Despite this, these results and those of others [23] show that an understanding of mosquito behaviour around the occupied bed net could inform bed net design in important ways. For example, higher levels of *An. gambiae* pressure around the bottom of the bed net suggest that reinforcement to make the net more hole-proof in this area could be an important improvement. Interestingly, a reinforced bottom strip is already a feature of PermaNet 3.0® bed nets. This strip may make holes less likely in this high-wear area though whether its inclusion was based on mosquito pressure considerations such as those described here is not known to the authors. This same design feature appears to be less called for in ITNs used to protect against *An. albimanus* though it is probably still justified in terms of the wear potential in this area of the net. Nonetheless, the total absence of any discussion about whether ITN design should be different for different mosquito species and different locales is indicative of the absence of considerations of mosquito behaviour in ITN design.

The bed net roof is much less vulnerable to holes than the bottom of the net but accumulates some [15] and, as these results show, roof holes should not be ignored since this area of the net is under potentially great mosquito pressure. Reinforcing the roof panel might be an effective way to improve ITN performance for both species in this study. Vestergaard-Frandsen PermaNet 3.0® bed nets already use 100 denier polyethylene thread in the roof panel (compared to the thinner 75 denier polyester thread of PermaNet 2.0 nets). The heavier fibre in the roof panel of PermaNet 3.0 nets may provide greater resistance to hole damage in the area where it would have the greatest impact.

While ongoing design changes are important to continue to improve bed net performance and durability, it is also important to keep in mind that changes made for one reason (e.g., increased durability) may have unintended effects in other areas (e.g., mosquito behaviour). von Seidlein *et al.*[39] show that bed net mesh density correlates positively with the degree of air flow attenuation inside and across the net. This will affect the dynamics and flow of the convective plume. Making roof panels more durable with heavier materials could, as a result, create additional resistance to movement of the convective plume through the roof, perhaps redirecting some of it through the net sides and ends. In turn, this could have the unintended effect of changing the patterns of mosquito pressure on the net, perhaps partly or fully negating the benefit of the original change. Changes in the amounts or types of insecticide incorporated in the ITN should also be considered carefully and in light of their known or expected effects on mosquito behavior. These results indicate that deltamethrin at the levels incorporated in PermaNet 2.0® bed nets did not reduce the amount of contact of the mosquitoes with treated surfaces. It is not known, however, how increased levels of deltamethrin or how other insecticides such as permethrin, or other components such as the pyrethroid synergist piperonyl butoxide that is incorporated into PermaNet 3.0 ITNs, may affect contact time and therefore unintentionally affect lethality and knockdown.

Potential effects of bed net design changes on mosquito behaviour are many, could have great public health and programme cost implications and cannot necessarily be predicted given the current rudimentary understanding of mosquito host-seeking interactions around ITNs.

## Conclusion

In real life, people sleep in all sorts of positions and clothing, with various amounts of bedding, change positions through the night and they often sleep under the same bed net with others. In addition, various types and sizes of bed nets are in use, they are hung in different ways and are set up in a wide range of situations in people’s homes. These experiments deal with a single highly simplified set of these variations since only one type of bed net was used and it was set up on a frame with all sides exposed. In addition, subjects dressed alike, were alone under the bed net, lay on their backs without sheets or blankets and with bare legs extended and arms at their sides. While additional experiments testing the effects of some of the variations listed above may be helpful (e.g., do mosquitoes fly above the net roof more if one side of the bed net is against a wall?), accounting for a significant number of them and their various interactions would be a daunting task and perhaps an unnecessary one if a complete understanding of mosquito responses around the bed (such as and improved and refined version of the model presented in Figure 17) can be developed. Such an understanding would allow the effects of the variations listed above (and others) on mosquito behaviour to be understood from first principles as opposed to at a level that would treat each situation as unique. To advance this approach, work is underway testing various hypotheses suggested by the model. This involves making systematic observations of the environmental factors in the tent and close to different parts of the occupied bed net (e.g., temperature, humidity, CO_2_ concentration, host odours, etc.) and doing experimental manipulations (e.g., augmenting CO_2_ levels in the tent with a tanked source, adding to or reducing heat and moisture components of the convective plume, introducing cross-draughts to change the direction and structure of the convective plume, etc.).

Curtis *et al.*[11] aptly liken ITNs to “… [mosquito] traps baited by the body odour of the occupant…”. Given that hundreds of millions of these human-baited traps have been deployed worldwide, it seems surprising that their performance in terms of the mosquito behaviour they are meant to thwart has been allowed to be largely absent from the discussion of ITN design, deployment and monitoring. Work like that presented here provides a useful contribution in this area and illustrates that much more needs to be done. Areas in need of additional attention include, but are not limited to: 1) ‘whole net’ type studies on other anopheline species to build up a picture of the range of behavioural patterns exhibited around bed nets; 2) field validation with wild mosquitoes of laboratory results such as those reported here; 3) development of conversion rates relating mosquito pressure measurements to probabilities of hole entrance; and, 4) studies of how decreasing insecticide content and/or increasing mosquito resistance affect interactions of mosquitoes with bed nets. An additional much-needed piece of the puzzle is how hole size and orientation (vertical as on net sides and ends or horizontal as on the roof) influence hole passage by the mosquito.

## Endnotes

^a^The following reagent was obtained through the MR4 as part of the BEI Resources Repository, NIAID, NIH: *Anopheles albimanus* STECLA [Santa Tecla], MRA-126, deposited by MQ Benedict. For additional information, see http://mr4.org/MR4ReagentsSearch/livingMosquitoes/MRA-126.aspx

^b^Cone tests performed by placing five mosquitoes from applicable colonies into each of three cones attached to side, end and top of treated and untreated bed nets. In all cases, knockdown after one hour, and lethality after 24 hr was 100% for treated bed nets and 0% for untreated bed nets.

## Abbreviations

ITN: Insecticide-treated bed net; LLIN: Long-lasting, insecticide-treated bed net; CO2: Carbon dioxide; MR4: Malaria Research and Reference Resource Center; CDC: United States Centers for Disease Control and Prevention; HC: Hierarchical cluster analysis; RH: Relative humidity; pHI: Proportionate hole index; FA: Functional area; WF: Weighting factor.

## Competing interests

The authors have declared that they have no competing interests.

## Authors’ contributions

JS conceived and planned the study, supervised all experiments and took the lead in manuscript preparation. SY conducted all statistical analyses and took part in manuscript preparation. Both authors read and approved the final manuscript.
